# Difference in persistent tuberculosis bacteria between in vitro and sputum from patients: implications for translational predictions

**DOI:** 10.1038/s41598-020-72472-y

**Published:** 2020-09-23

**Authors:** Alan Faraj, Oskar Clewe, Robin J. Svensson, Galina V. Mukamolova, Michael R. Barer, Ulrika S. H. Simonsson

**Affiliations:** 1 Department of Pharmaceutical Biosciences, Uppsala University, Uppsala, Sweden; 2 Leicester Tuberculosis Research Group, Department of Respiratory Sciences, University of Leicester, Leicester, UK; 3 Department of Microbiology, University of Leicester, Leicester, UK

**Keywords:** Tuberculosis, Biomarkers, Pharmacodynamics

## Abstract

This study aimed to investigate the number of persistent bacteria in sputum from tuberculosis patients compared to in vitro and to suggest a model-based approach for accounting for the potential difference. Sputum smear positive patients (n = 25) provided sputum samples prior to onset of chemotherapy. The number of cells detected by conventional agar colony forming unit (CFU) and most probable number (MPN) with Rpf supplementation were quantified. Persistent bacteria was assumed to be the difference between MPN_rpf_ and CFU. The difference in persistent bacteria between in vitro and human sputum prior to chemotherapy was quantified using different model-based approaches. The persistent bacteria in sputum was 17% of the in vitro levels, suggesting a difference in phenotypic resistance, whereas no difference was found for multiplying bacterial subpopulations*.* Clinical trial simulations showed that the predicted time to 2 log fall in MPN_rpf_ in a Phase 2a setting using in vitro pre-clinical efficacy information, would be almost 3 days longer if drug response was predicted ignoring the difference in phenotypic resistance. The discovered phenotypic differences between in vitro and humans prior to chemotherapy could have implications on translational efforts but can be accounted for using a model-based approach for translating in vitro to human drug response.

## Introduction

Treatment of tuberculosis (TB) is today consisting of an extensive treatment duration with a combination of several drugs^1^. Shortening the time to treatment success is a pillar in TB research in order to improve the quality of life for patients, increase adherence and potentially improve relapse rates. The latter is an ambition that is likely to depend on drugs capability to act on hard-to-kill persisters^2^. One part of the current efforts in drug development against TB focuses on the relevance of drug effect on persisters when generating candidate drugs for further development^3^. Due to the expected importance of these phenotypically resistant bacteria in treatment of TB, it is crucial to determine an experimental drug’s efficacy not only on multiplying bacteria, but on persisters as well. It is important that research efforts that are initiated utilize informative pre-clinical experimental methods and analyze the generated data in an innovative manner. Usage of exposure–response models is an informative way of incorporating information from multiple sources, such as pharmacokinetics and data from one or several biomarkers into one analysis. As the biomarkers tell different stories about the status of disease, models incorporating different biomarkers are important to make informed decisions^4^ and prospectively predict different experimental conditions.

**Figure 1:**
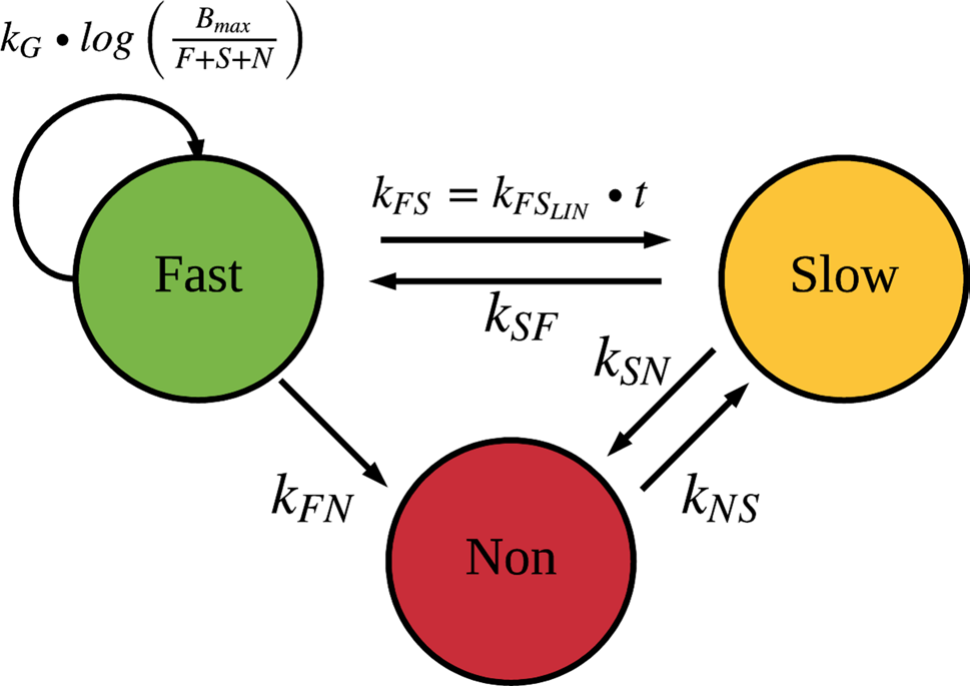
Schematic illustration of the Multistate Tuberculosis Pharmacometric model. F, fast-multiplying bacterial state; S, slow-multiplying bacterial state; N, non-multiplying bacterial state; k_G,_ growth rate of the fast-multiplying state bacteria; k_FS_, time-dependent linear rate parameter describing transfer from fast- to slow-multiplying bacterial state; k_SF_, first-order transfer rate between slow- and fast-multiplying bacterial state; k_FN_, first-order transfer rate between fast- and non-multiplying bacterial state; k_SN_, first-order transfer rate between slow- and non-multiplying bacterial state; k_NS_, first-order transfer rate between non-multiplying and slow-multiplying bacterial state. B_max_ is the system carrying capacity per ml sputum.

Originally developed using in vitro data, the Multistate Tuberculosis Pharmacometric (MTP) model^5^ describes the relationship between three subpopulations of *Mycobacterium tuberculosis*, a fast-, a slow- and a non-multiplying state (persisters) corresponding to difference in metabolic activity which the bacteria can switch between (Fig. 1). The framework has been applied to describe in vitro natural growth and drug effect data^5,6^, in vivo data^7^ and also on clinical trial data^8,9^ bridging exposure from a population pharmacokinetic model as for example rifampicin^10^ and biomarker data. As the model has ability to describe data from both pre-clinical and clinical phase of drug development, it has a role in translational efforts which previously have been demonstrated in a study that predicted rifampicin early bactericidal activity (EBA) efficacy data based on in vitro information and translational factors^11^. It has also been used as a show case example of important quantitative pharmacology work that can accelerate drug development^12^. Clinical trial simulation can also be used to evaluate potential difference in efficacy for subgroups such as renal impairment for drugs that are renally cleared^13^, the implication of concomitant food^14^ or polymorphism for drugs being given concomitant with a drug with enzyme inductive properties^15^. What differentiates the MTP model from other empirical in silico models^16^ is the incorporation of the persisters state denoted as non-multiplying bacteria. Bacteria are described as being able to transfer to and from the different substates and the change from a multiplying to a stationary phase culture in vitro allowed the quantification of the ratio of fast- and slow-multiplying bacteria to persistent bacteria in the original analysis based on in vitro CFU data^5^. It is not possible to directly quantify proportion of persistent bacteria in relation to the multiplying bacteria in humans using only the CFU biomarker since TB patients are only presented with symptoms when they are in the stationary phase.

Studies have shown that using a most probable number (MPN) assay in media supplemented with culture filtrate containing resuscitation promoting factors (rpfs) quantifies an occult population corresponding to persisters, which constitutes a majority of the total bacterial population^17^. In contrast to CFU counts that lack the ability to quantify subpopulations not able to grow on plates, assays such as MPN_rpf_ provide direct information on persisters. Note that CFU is able to inform about persisters indirectly for instance under assumptions of a model like the MTP model. By using a model-based analysis, it is possible to simultaneously analyze CFU and MPN_rpf_ counts to investigate how the biomarkers are related. Further, since the MPN_rpf_ biomarker quantifies persistent bacteria it could be used to investigate if the relative amount of persistent bacteria is the same in an in vitro system as in patients.

The aim of this analysis was to investigate if there is a difference in number of persistent bacteria between TB patients and in vitro and to suggest a model-based approach for accounting for the potential difference.

## Results

### Pharmacodynamic modeling

This study included simultaneous analysis of CFU and MPN_rpf_ biomarker data from 25 patients prior to treatment. The biomarker data was simultaneously analyzed with non-linear mixed effects modeling using the MTP model. Re-estimation of the system carrying capacity (B_max_) enabled for adjustment from the in vitro estimate to the magnitude of the biomarker quantity derived from sputum samples, provided by patients. In this way, the model accounted for that the inoculum in vitro is different from baseline biomarker in human. As such, the difference in persistent bacteria between in vitro and in human is not due to difference in baseline/inoculum. The addition of inter-individual variability (IIV) in B_max_ was statistically significant for all implementation approaches (∆OFV = 13.64, − 9.86, − 9.86 for Method 1, 2 and 3 respectively). IIV was not supported by the data when added on any of the initial bacterial number parameters.

**Figure 2:**
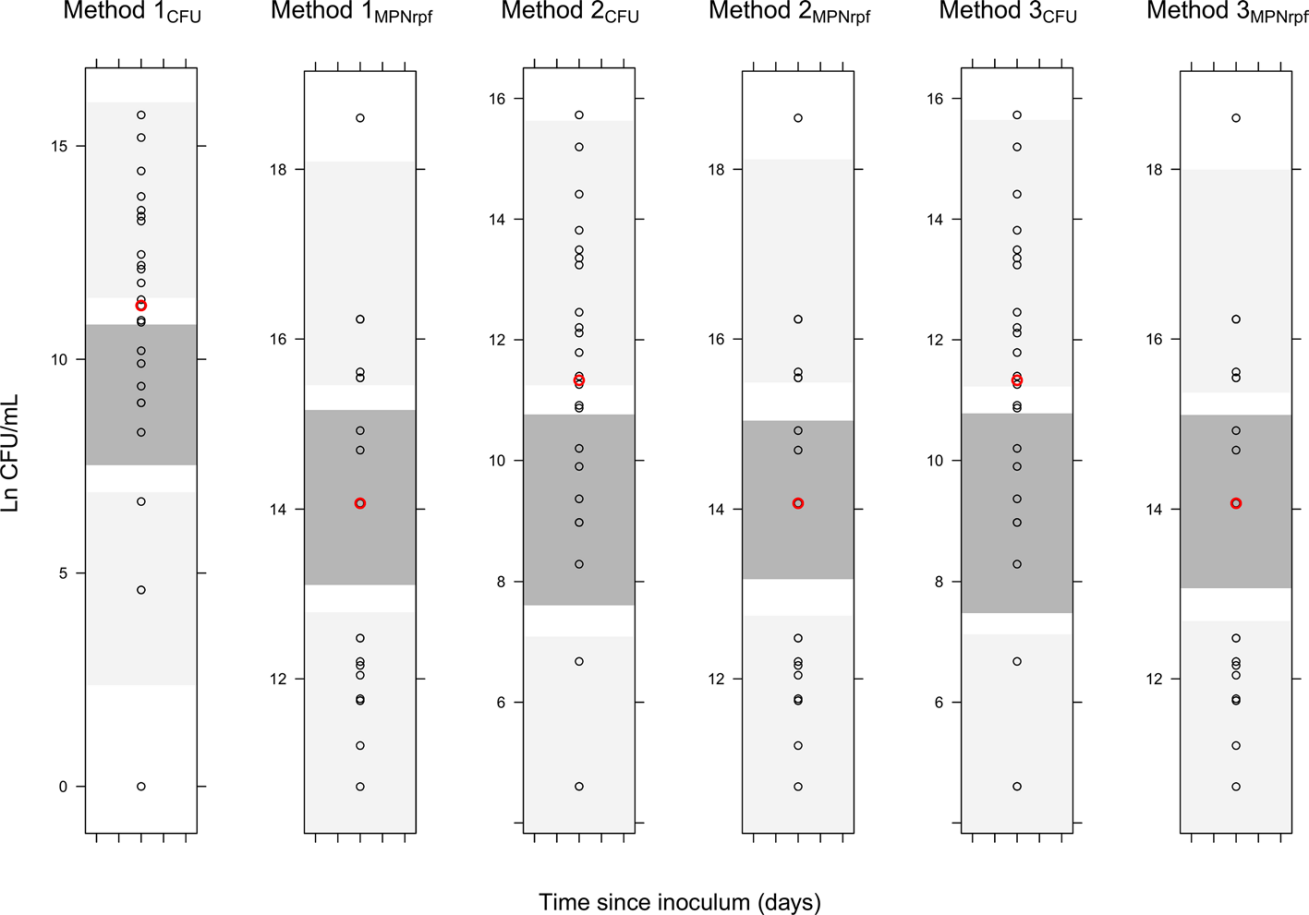
Visual predictive checks (VPCs) for the final models without the CCF. For each method to handle the predictions, human baseline CFU and MPN_rpf_ observed data (circles) and simulations (shaded areas) are displayed. From top to bottom, shaded areas represent 95% confidence intervals of the 90th (light grey), median (dark grey) and 10th (light grey) percentiles of simulated data based on 1,000 simulations. The red circle indicates the median of observed data.

**Figure 3:**
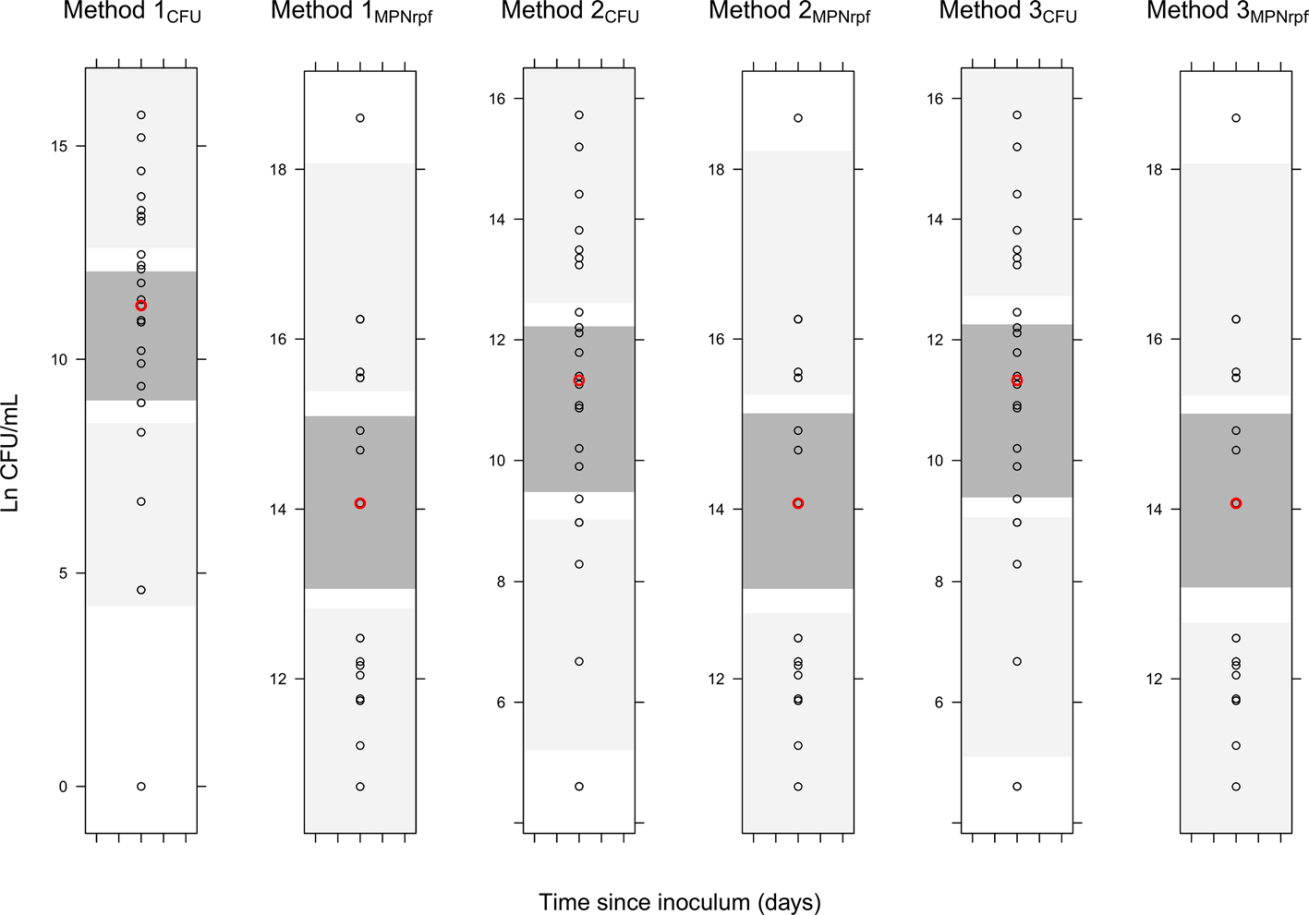
Visual predictive checks (VPCs) for the final models with the CCF. For each method to handle the predictions, human baseline CFU and MPN_rpf_ observed data (circles) and simulations (shaded areas) are displayed. From top to bottom, shaded areas represent 95% confidence intervals of the 90th (light grey), median (dark grey) and 10th (light grey) percentiles of simulated data based on 1,000 simulations. The red circle indicates the median of observed data.

The addition of a clinical conversion factor (CCF), adjusting the contribution of the persister bacterial sub-population to the total bacterial load (i.e. MPN_rpf_ quantity) in sputum relative to in vitro was statistically significant (∆OFV = − 7.07, − 12.74, − 12.74 for Methods 1, 2 and 3 respectively) using all different approaches to handle the predictions. CCF was not statistically significant for any of the other bacterial subpopulations, regardless of the different approaches to handle the predictions. The estimates of CCF from the different approaches to handle the predictions was 0.17, 0.18 and 0.24 for Method 1, 2 and 3, respectively (Table 1). Using method 1 for simplicity, this is to be interpreted as the human persistent bacteria was only 17% of what was predicted from a stationary in vitro culture. The model without CCF systematically under-predicted the CFU data (Fig. 2) whereas the final models including CCF successfully described both CFU and MPN_rpf_ data, as seen in Fig. 3. All final parameters are presented in Table 1.

**Table 1:** Parameter estimates of the final Multistate Tuberculosis Pharmacometric (MTP) model applied to CFU and MPN_rpf_ data.

Parameters	Description	Population estimate	%RSE
**MTP model parameters**			
Fixed effects			
k_G_ (days^−1^)	Fast-multiplying bacterial growth rate	0.206 FIX	–
k_FN_ (days^−1^)	Transfer rate from fast- to non-multiplying state	8.98·10^–7^ FIX	–
k_SN_ (days^−1^)	Transfer rate from slow- to non-multiplying state	0.186 FIX	–
k_SF_ (days^−1^)	Transfer rate from slow- to fast-multiplying state	0.0145 FIX	–
k_NS_ (days^−1^)	Transfer rate from non- to fast-multiplying state	0.00123 FIX	–
k_FSLin_ (days^−2^)	Time-dependent transfer rate from fast- to slow-multiplying state	0.00166 FIX	–
F_0_ (ml^−1^)	Initial bacterial number of fast-multiplying state	4.11 FIX	–
S_0_ (ml^−1^)	Initial bacterial number of slow-multiplying state	9,770 FIX	–
B_max_ (ml^−1^)^a^	System carrying capacity per ml sputum in human	5.54·10^6^	71.3
B_max_ (ml^−1^)^b^	System carrying capacity per ml sputum in human	7.47·10^6^	59.7
B_max_ (ml^−1^)^c^	System carrying capacity per ml sputum in human	2.68·10^6^	41.8
CCF^a^	Persistent translational factor	0.24	51.3
CCF^b^	Persistent translational factor	0.17	43.8
CCF^c^	Persistent translational factor	0.19	14.2
Random effects			
IIV in B_max_^a^ (%CV)	Inter-individual variability of B_max_	206	9.88
IIV in B_max_^b^ (%CV)	Inter-individual variability of B_max_	206	9.88
IIV in B_max_^c^ (%CV)	Inter-individual variability of B_max_	206	9.88
Residual error parameters			
Add CFU^a^	Additive error of CFU prediction	250	17.4
Add MPN_rpf_^a^	Additive error of MPN_rpf_ prediction	1.90·10^–4^	17.8
Add CFU^b^	Additive error of CFU prediction	206	15.5
Add MPN_rpf_^b^	Additive error of MPN_rpf_ prediction	1.90·10^–4^	300
Add CFU^c^	Additive error of CFU prediction	207	15.4
Add MPN_rpf_^c^	Additive error of MPN_rpf_ prediction	1.90·10^–4^	6.70
**Drug pharmacokinetic parameters**^**d**^			
CL/F (L/h)	Oral clearance	8.00	–
V/F (L)	Apparent volume of distribution	60.0	–
k_a_ (h^−1^)	Absorption rate constant	1.00	–
**Exposure–response parameters**			
Killing of fast-multiplying state			
FD_k_ (L mg^−1^ days^−1^)	Second-order fast-multiplying state death rate	0.33	–
Killing of slow-multiplying state			
SD_k_ (L mg^−1^ days^−1^)	Second-order slow-multiplying state death rate	0.33	–
Killing of non-multiplying state			
ND_k_ (L mg^−1^ days^−1^)	Second-order non-multiplying state death rate	0.33	–
Inhibition of fast, killing of slow and non-multiplying bacteria			
FG_on/off_	Fractional inhibition of growth of fast-multiplying state	1.00	–
SD_k_ (L mg^−1^ days^−1^)	Second-order slow-multiplying state death rate	0.30	–
ND_k_ (L mg^−1^ days^−1^)	Second-order non-multiplying state death rate	0.31	–

^a^Parameter values from implementation method 1 as defined in the materials and methods section.

^b^Parameter values from implementation method 2 as defined in the materials and methods section.

^c^Parameter values from implementation method 3 as defined in the materials and methods section.

^d^The pharmacokinetic parameters were the same for all hypothetical drugs. FIX, the parameter was fixed according to^5^. RSE, relative standard error as obtained from the covariance step in NONMEM.

The results suggest to include CCF as a translational factor accounting for the difference in amount of persister mycobacteria in clinical MPN_rpf_ sputum samples from patients relative to in vitro. The biomarker MPN_rpf_ includes quantitative information on persister mycobacteria that remains undetected using CFU as a biomarker. The final model was defined as in Eqs. (1), (2) and (3) where each differential equation describes the dynamics of fast-multiplying, slow-multiplying and persister mycobacteria, respectively. The simultaneous analysis of CFU and MPN_rpf_ data was performed using the sputum sampling compartment method as following:

1 $$\frac{d{Sample}_{CFU}}{dt}={k}_{production}\cdot \left(F+S\right)$$

2 $$\frac{d{Sample}_{MPN-{Rpf}_{CCF}}}{dt}={k}_{production}\cdot (F+S+(CCF*N))$$

in which the sputum production rate (k_production_) was given from ${k}_{production}= \frac{{Volume}_{sputum}}{{Duration}_{sampling}}$ (mL/h). Finally, the model predictions of CFU and MPN_rpf_ for TB patients prior to treatment were handled as following:

3 $$PRED=\mathrm{log}\left(\frac{Sample}{{Volume}_{sputum}}\right)$$

where volume denotes the collected volume of sputum during a collection interval.

The percentage of persisters in human sputum prior to treatment in relation to total bacterial amount ranged from 96 to 97% in Methods 1, 2 and 3, compared to 99% in vitro predicted at day 150 in a stationary culture. The relative percentage of persisters to total bacteria was similar between in vitro and in humans due to the large proportion of persisters in both systems.

**Figure 4:**
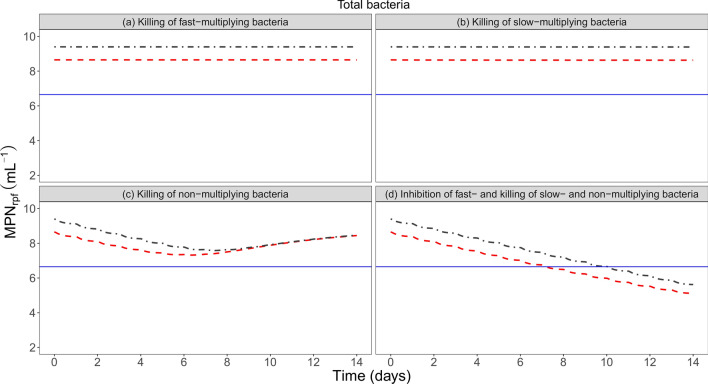
Typical predictions of log10 MPN_rpf_ in human using only in vitro information without accounting for the clinical conversion factor (CCF) (dark grey) and in human using in vitro information and accounting for CCF (red) after hypothetical killing of (**a**) fast-, (**b**) slow- and (**c**) non-multiplying bacteria, and (**d**) combination effect including inhibition of fast-multiplying bacteria and killing of slow- and non-multiplying bacteria. The blue horizontal line illustrates a 2 log fall threshold in MPN_rpf_ when accounting for the CCF.

**Figure 5:**
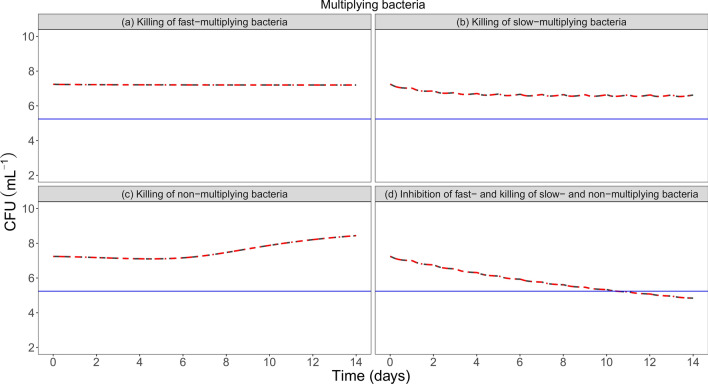
Typical predictions of log10 CFU in human using only in vitro information without accounting for the clinical conversion factor (CCF) (dark grey) and in human using in vitro information and accounting for CCF (red) after hypothetical killing of (**a**) fast-, (**b**) slow- and (**c**) non-multiplying bacteria, and (**d**) combination effect including inhibition of fast-multiplying bacteria and killing of slow- and non-multiplying bacteria. The blue horizontal line illustrates a 2 log fall threshold in CFU when accounting for the CCF.

Clinical trial simulations of four different hypothetical drugs with similar PK showed direct consequences of the findings in this work for a biomarker that depicts total bacteria, as MPN_rpf_. For a drug that in combination inhibits the growth of fast multiplying bacteria and kills slow- and non-multiplying bacteria, the predicted time to 2 log fall in total bacterial number (MPN_rpf_) in human based on only in vitro information with and without accounting for the CCF is shown in Fig. 4. On a typical level, the predicted time to 2 log fall in MPN_rpf_ in human was almost 3 days shorter when accounting for the difference in phenotypic resistance between in vitro and human. For a drug only killing fast- slow- or non-multiplying bacteria, there was no difference in predicted CFU and as such no impact of difference in phenotypic resistance on human CFU readouts (Fig. 5).

## Discussion

We show in this work using both CFU and MPN biomarker data, that the persistent bacteria in human sputum was only 17% of what is predicted from a stationary in vitro culture prior to chemotherapy, i.e. there is a difference in phenotypic resistance between in vitro and human*.* There was no difference in the number of multiplying bacteria, implying no difference in the CFU readouts when predicting a drug effect in human from in vitro (Fig. 5), as CFU is informed by multiplying bacteria. However, for more informative biomarkers like MPN_rpf_ which contains information on total bacteria, the lower number of persisters in human compared to in vitro is visible in the biomarker readouts, prior to chemotherapy (Fig. 4)*.* Such differences will have direct effect on time to eradication, happening earlier in human than predicted from in vitro when ignoring the CCF (Fig. 4), and can be taken in to account by the developed model*.* Unless accounted for the difference, the predictions of human treatment length will be longer as persisters most often is the bacterial population which is most difficult to kill and will remain in the patients at the end of treatment when the CFU readout is negative. This can be seen for a drug with combination effect on different subpopulations in the simulations (Fig. 4) revealing that the predicted time to 2 log fall in MPN_rpf_ would take almost 3 days longer if drug response was predicted from in vitro preclinical information but ignoring difference in phenotypic resistance between human and in vitro. This have implications for drug development as many of the new regimens will focus on shortening current treatment lengths. For the hypothetical drugs killing fast-, slow- or non-multiplying bacteria, low EBA activity is predicted as described by MPN_rpf_ (Fig. 4) and CFU (Fig. 5). This is in line with EBA results for drugs killing slow- or non-multiplying bacteria, like pyrazinamide and clofazimine, respectively^9,18^.

In this work, we did not have experimental data on potential difference between in vitro and human with respect to persisters during treatment. As such, the simulations are assuming that the drug effect is similar between in vitro and human regardless of the difference in initial levels of persisters. Future studies should evaluate potential difference in treatment effect between in vitro and humans with respect to persisters for instance by including longitudinal biomarker data on persisters (for instance MPNrpf counts) in addition to CFU counts. Initial bacterial burden have earlier been shown to be a predictor for treatment response^19^. As such, a difference in initial bacterial level of persisters could potentially result in a difference in treatment effect on persisters. Despite the difference in levels of persisters between in vitro and humans, the persisters will remain the major sub-population both in vitro and in humans. The identified difference in phenotypic resistance can be accounted for in a model-based translational framework^11^. To show this we used samples from sputum smear positive patients that was collected prior to onset of chemotherapy, followed by mycobacterial quantification using CFU, and MPN counts treated with RPFs^17^. We described these data with a previously developed model, the MTP model, predicting different bacterial sub-populations and compared the human subpopulation predictions to model predicted in vitro bacterial sub-states. In order to translate in vitro drug efficacy to human, the relative ratio of persisters to total bacteria needs to be accounted for. We propose that a conversion factor is used when translating from in vitro to humans. In order to facilitate this, we present three different implementation strategies (Methods 1–3) that can be employed dependent on the data, for instance with the sputum sampling compartment in an clinical setting^8^. The translational factor was evaluated on all three different subpopulations and found to only be supported (compared to a model without the factor) for the persistent subpopulation.

The three the different approaches to handle the predictions resulted in similar results. The value of the CCF ranged from 0.17 to 0.24, which implies that 17–24% of the in vitro predicted amount of persisters appear in sputum. That corresponds to a situation where the typical ratio of persister mycobacteria to total bacterial load ranges from 96 to 97% compared to 99% without the translational factor, in sputum at 150 days after inoculum. The reason for the difference in persisters between in vitro and patients is not known but could be due to lung barrier preventing persisters that are within lesions to be coughed up in sputum. Alternatively, the statistically significant translational factor could be due to the fact that on solid media, bacteria grow in absence of an immune system whereas in humans it is likely that the growth is controlled. The relationship between persisters to total bacterial number prior and post chemotherapy is of importance in translational efforts to measure treatment response in a more representative manner of TB which should be studied further in simulation studies. Further investigation is encouraged analysing MPN_rpf_ and CFU counts over time not only prior to chemotherapy but also during treatment in patients, as the ratio of persisters to total bacterial burden may vary more over time and treatment and the presented model can be used to model this relationship.

In order to account for differences in magnitude of bacterial load between the in vitro and human sputum samples, B_max_ was re-estimated. Further, adjusting for differences between individuals with respect to CFU and MPN_rpf_ counts, an IIV parameter was found to be statistically significant. Independent of approach to handle the predictions, a CCF was statistically significant which enabled adjustment of the predicted persister bacteria contribution to the predicted total biomarker quantity in TB patients (i.e. MPN_rpf_). As the re-estimation of B_max_ only affects the magnitude of bacterial load and not the model-predicted relative amounts of the different bacterial states, the predictions without the translational factor is solely based on CFU with respect to relative amounts of the different bacterial sub-states. Thus, the introduction of the translational factor enabled adjustment of the relative amounts of multiplying and non-multiplying bacterial sub-states to the relative amounts derived based on CFU data only. Further, by informing the model with both biomarkers simultaneously, the flexibility of the MTP model system can be used to describe clinical data without re-estimation of the system parameters related to transfer between bacterial states. As the transfer rates governs the relative ratio between the multiplying, slow-multiplying and persister sub-states, the introduction of the translational factor enabled adjustment of relative ratio of the sub-states while keeping the semi-mechanistic structure of the model. It is desirable to be able to adjust for different relative amounts of bacterial sub-states without re-estimation of system parameters in absence of data, as studying natural growth of TB in humans is not ethical or plausible.

The model-based analysis of human sputum CFU and MPN_rpf_ in relation to the in vitro predicted relative bacterial amounts identified differences in relative ratio of the persister subpopulation between in vitro and in sputum from TB patients. This allowed development of three different implementations of a model-based translational approach for accounting for this difference when predicting human CFU and/or MPN_rpf_ based on in vitro information. As different biomarkers tell different stories about disease and bacterial burden, it is beneficial to utilize frameworks that can analyse more than one biomarker simultaneously over time as an alternative to develop new biomarkers. Thus, the developed strategy has potential to increase understanding of TB disease status under treatment and possibly have impact on decision-making of which candidate drugs to reject or advance in preclinical and clinical development.

With this strategy we discovered phenotypic differences between in vitro and humans prior to chemotherapy, which could have implications of translational efforts. The identified difference between human sputum and in vitro can be accounted for using a model-based approach for translating in vitro to human drug response.

## Materials and methods

### Patients and study design

The study was approved by Leicestershire, Northamptonshire, and Rutland Research Ethics Committee (07/Q2501/58) and was conducted in line with the Declaration of Helsinki. Sputum smear positive patients (n = 25) provided baseline sputum samples prior to onset of chemotherapy, after informed consent was obtained. The number of cells detected by conventional agar CFU and MPN with and without Rpf supplementation were quantified. Both MPN and CFU growth assays were performed in quadruple replicates (11). Raw data from all 25 patients can be found in supplementary materials of the original publication^17^. In this work, CFU and MPN_rpf_ data were used. More information on sample handling protocols can be found in the original study^17^.

### Pharmacodynamic modeling

Observed bacterial numbers from both CFU and MPN_rpf_ assays were transformed to natural logarithms. The MTP model^5^ (Fig. 1), initially developed on in vitro data but used to describe in vivo^7^ and clinical data^8,11^*,* was utilized to predict the bacterial numbers of different bacterial subpopulations in vitro and in human, corresponding to multiplying (F), semi-dormant (S) and persistent (N) mycobacteria 150 days after inoculum. The following differential equations defined the MTP model:

4 $$\frac{\mathrm{dF}}{\mathrm{dt}}={\mathrm{k}}_{\mathrm{G}}\cdot \mathrm{log}\left(\frac{{\mathrm{B}}_{\mathrm{max}}}{\mathrm{F}+\mathrm{S}+\mathrm{N}}\right)\cdot \mathrm{F}+{\mathrm{k}}_{\mathrm{SF}}\cdot \mathrm{S}-{\mathrm{k}}_{\mathrm{FS}}\cdot \mathrm{F}-{\mathrm{k}}_{\mathrm{FN}}\cdot \mathrm{F}$$

5 $$\frac{dS}{dt} ={k}_{FS}\cdot F+{k}_{NS}\cdot N-{k}_{SN}\cdot S-{k}_{SF}\cdot S$$

6 $$\frac{dN}{dt}={k}_{SN}\cdot S+{k}_{FN}\cdot F-{k}_{NS}\cdot N$$

Transfer rates was denoted with two-letter subscripts referring to the origin and direction, respectively, where ${k}_{FS}={k}_{FSlin}\cdot t$ was unique for time after infection (days) dependency. The flows were assumed to mirror change in metabolic activity. The parameter k_G_ was defined as the growth rate of multiplying bacteria and B_max_ as the carrying capacity in the system, limiting the growth in stationary phase. To adjust the model-predicted magnitude of bacterial number given the clinical data, B_max_ was estimated using both CFU and MPN_rpf_ data. An IIV parameter was tested for B_max_ and the initial number of each bacterial sub-state at inoculums (assumed to occur 150 days prior to the sampling time-point). The predicted bacterial number (ml^−1^) of fast-multiplying, slow-multiplying and persistent bacteria was defined as the distinct quantity in each compartment presented above. The difference in relative ratio of different bacterial subpopulations in human compared to in vitro, prior to chemotherapy, was predicted.

CFU and MPN_rpf_ data were simultaneously analysed and the difference in persisters between in vitro and human was handled using three different approaches. First, a previously developed approach (Method 1) was applied, the sputum sampling compartment method^8^. Model-prediction of CFU was defined as average sum of multiplying and slow-multiplying (semi-dormant bacterial) number (ml^−1^) within the sputum collection time interval and the prediction of MPN_rpf_ as the average sum of multiplying, semi-dormant and persistent bacterial number (ml^−1^), in the assumed sputum sampling interval of 15 min. This approach required inclusion of a sputum sample compartment defined for CFU as:

7 $$\frac{d{Sample}_{CFU}}{dt}={k}_{production}\cdot (F+S)$$

and for MPN:

8 $$\frac{d{Sample}_{MPN-Rpf}}{dt}={k}_{production}\cdot (F+S+N)$$

where ${k}_{production}= \frac{{Volume}_{sputum}}{{Duration}_{sampling}}$ (mL/h). A sample was defined as the bacterial number in a volume (mL) that was collected over the assumed duration of 15 min described by the sampling interval. Prior to start of each individuals sampling interval, the amount in the sample compartment was set to 0. Predictions of CFU and MPN_rpf_ were defined as:

9 $$PRED=\mathrm{log}\left(\frac{Sample}{{Volume}_{sputum}}\right)$$

in which PRED is the natural logarithm of the average bacterial number (ml^−1^) over the sampling interval of fast-multiplying and slow-multipying bacteria for CFU, and fast-multiplying, slow multipying and persistent bacteria for MPN_rpf_. The sample volume was assumed to be 5 mL, which is a plausible value based on a previous report^20^. Both the duration of the sampling interval and the volume collected was specified in the dataset.

Apart from estimating B_max_ to adjust the model-predicted biomarker magnitude, a clinical translational factor was estimated to adjust for each of the model-predicted bacterial sub-populations contribution, to the model-predicted biomarker quantity in sputum. The translational factor was evaluated on the model-prediction of the fast-multiplying, slow-multiplying and persistent state, estimating the percentage of a given bacterial subtype in sputum in relation to the MTP model-predicted number, based on in vitro estimates. The CCF was introduced one at the time as follows:

10 $$\frac{d{Sample}_{MPN-{Rpf}_{CCF}}}{dt}={k}_{production}\cdot ((CCF*F)+S+N)$$

11 $$\frac{d{Sample}_{MPN-{Rpf}_{CCF}}}{dt}={k}_{production}\cdot (F+(CCF*S)+N)$$

12 $$\frac{d{Sample}_{MPN-{Rpf}_{CCF}}}{dt}={k}_{production}\cdot (F+S+(CCF*N))$$

Adjusting the fast-multiplying, slow-multiplying and persistent bacteria (i.e. Rpf-dependent bacilli) contribution to the model-predicted MPN_rpf_ biomarker quantity in sputum, respectively.

In the second approach used for evaluation (Method 2), the predictions of the different bacterial subpopulations were defined as the predicted bacterial number (ml^−1^) in the fast-multiplying and slow-multiplying compartments for CFU, whereas for MPN, the predictions were defined as the bacterial number (ml^−1^) in all states:

13 $${PRED}_{CFU}=\mathrm{log}(F+S)$$

14 $${PRED}_{MPN-Rpf}=\mathrm{log}(F+S+N)$$

In addition to re-estimation of B_max_, CCF was estimated in the same fashion as for the sputum sampling compartment method. The parameter was constrained to be positive and was implemented one at the time as follows:

15 $${PRED}_{MPN-Rpf}=\mathrm{log}((CCF*F)+S+N)$$

16 $${PRED}_{MPN-Rpf}=\mathrm{log}(F+(CCF*S)+N)$$

17 $${PRED}_{MPN-Rpf}=\mathrm{log}(F+S+(CCF*N))$$

Adjusting for each model-predicted bacterial subpopulations contribution to the total model-predicted MPN_rpf_ biomarker quantity.

In the third approach used for evaluation (Method 3), the CCF was estimated on the differential equations describing the dynamics in the compartment of each bacterial subpopulation. The CCF was constrained to be positive and was introduced one at the time as following:

18 $$\frac{\mathrm{dF}}{\mathrm{dt}}=\left({\mathrm{k}}_{\mathrm{G}}\cdot \mathrm{log}\left(\frac{{\mathrm{B}}_{\mathrm{max}}}{\mathrm{F}+\mathrm{S}+\mathrm{N}}\right)\cdot \mathrm{F}+{\mathrm{k}}_{\mathrm{SF}}\cdot \mathrm{S}-{\mathrm{k}}_{\mathrm{FS}}\cdot \mathrm{F}-{\mathrm{k}}_{\mathrm{FN}}\cdot \mathrm{F}\right) \cdot CCF $$

19 $$\frac{dS}{dt} =({k}_{FS}\cdot F+{k}_{NS}\cdot N-{k}_{SN}\cdot S-{k}_{SF}\cdot S)\cdot CCF$$

20 $$\frac{dN}{dt}=({k}_{SN}\cdot S+{k}_{FN}\cdot F-{k}_{NS}\cdot N)\cdot CCF$$

Adjusting for the model-prediction of each bacterial subpopulation to the total biomarker quantity, which was defined for CFU as:

21 $${PRED}_{CFU}=\mathrm{log}(F+S)$$

and for MPN as:

22 $${PRED}_{MPN-Rpf}=\mathrm{log}(F+S+N)$$

The different implementation methods to handle the predictions for difference in persistent bacteria between in vitro and humans were subsequently used to predict the different bacterial subpopulations at 150 days after inoculum. The ratio of persisters to total bacteria for in vitro and in human was calculated as follows:

23 $$Ratio\,persister\,to\,total\,bacteria= \frac{Predicted\,number\,of\,persisters}{Predicted\,total\,bacterial\,number}$$

Simulations was performed to investigate the consequences of the results in an EBA trial setting. The typical total bacterial number (MPN_rpf_) and CFU was predicted in human from in vitro with and without the translational factor. The four different hypothetical scenarios of drug effect were killing of (a) fast, (b) slow, (c) non-multiplying bacteria as mono-effects and (d) inhibition of fast, killing of slow and non-multiplying bacteria as a combination effect (Table 1). The pharmacokinetic parameters for the hypothetical drugs was assumed to reflect a one-compartment disposition model with first order absorption and rapid elimination (Table 1).

### Statistical analysis

Modeling and simulation were performed using NONMEM (version 7.4; Icon Development Solutions, Elliot City, MD, USA)^21^. A hierarchical model with addition of one parameter was considered statistically significant at a 5% significance level if ∆OFV (calculated by NONMEM, as proportional to − 2 $x$ log-likelihood of the data) reduced by 3.84 for 1 degree of freedom (χ^2^ distribution). Visual predictive checks (VPCs) was performed to assess the predictive performance of the model, describing the 5th, median (50th) and 95th percentiles of the biomarker data (*n* = 1,000 simulations). All model diagnostics were conducted using Xpose and PsN^22,23^.

## Data Availability

The datasets generated during and/or analyzed during the current study are available from the corresponding author on reasonable request.

## References

[1] WHO. Guidelines for treatment of tuberculosis. WHO. https://www.who.int/tb/publications/2010/9789241547833/en/.

[2] Hu Y (2015). High-dose rifampicin kills persisters, shortens treatment duration, and reduces relapse rate in vitro and in vivo. Front. Microbiol..

[3] Coates ARM, Hu Y (2008). Targeting non-multiplying organisms as a way to develop novel antimicrobials. Trends Pharmacol. Sci..

[4] Svensson RJ (2019). Model-based relationship between the molecular bacterial load assay and time to positivity in liquid culture. Antimicrob. Agents Chemother..

[5] Clewe O, Aulin L, Hu Y, Coates ARM, Simonsson USH (2016). A multistate tuberculosis pharmacometric model: a framework for studying anti-tubercular drug effects in vitro. J. Antimicrob. Chemother..

[6] Clewe O, Wicha SG, de Vogel CP, de Steenwinkel JEM, Simonsson USH (2018). A model-informed preclinical approach for prediction of clinical pharmacodynamic interactions of anti-TB drug combinations. J. Antimicrob. Chemother..

[7] Chen C (2017). Assessing pharmacodynamic interactions in mice using the multistate tuberculosis pharmacometric and general pharmacodynamic interaction models. CPT Pharmacomet. Syst. Pharmacol..

[8] Svensson R, Simonsson U (2016). Application of the multistate tuberculosis pharmacometric model in patients with rifampicin-treated pulmonary tuberculosis. CPT Pharmacomet. Syst. Pharmacol..

[9] Faraj A, Svensson RJ, Diacon AH, Simonsson USH (2020). Drug effect of clofazimine on persisters explain an unexpected increase in bacterial load from patients. Antimicrob. Agents Chemother..

[10] Svensson RJ (2018). A population pharmacokinetic model incorporating saturable pharmacokinetics and autoinduction for high rifampicin doses. Clin. Pharmacol. Ther..

[11] Wicha SG (2018). Forecasting clinical dose-response from preclinical studies in tuberculosis research: translational predictions with rifampicin. Clin. Pharmacol. Ther..

[12] Gupta, N. *et al.* Transforming translation through quantitative pharmacology for high impact decision-making in drug discovery and development. Accepted (2019). 10.1002/cpt.1667 31709519

[13] Jönsson S, Simonsson USH, Miller R, Karlsson MO (2015). Population pharmacokinetics of edoxaban and its main metabolite in a dedicated renal impairment study. J. Clin. Pharmacol..

[14] Zvada SP (2010). Effects of four different meal types on the population pharmacokinetics of single-dose rifapentine in healthy male volunteers. Antimicrob. Agents Chemother..

[15] Mihara K (1999). Stereospecific analysis of omeprazole supports artemisinin as a potent inducer of CYP2C19. Fundam. Clin. Pharmacol..

[16] Davies GR, Brindle R, Khoo SH, Aarons LJ (2006). Use of nonlinear mixed-effects analysis for improved precision of early pharmacodynamic measures in tuberculosis treatment. Antimicrob. Agents Chemother..

[17] Mukamolova GV, Turapov O, Malkin J, Woltmann G, Barer MR (2010). Resuscitation-promoting factors reveal an occult population of tubercle bacilli in sputum. Am. J. Respir. Crit. Care Med..

[18] Diacon AH (2015). Bactericidal activity of pyrazinamide and clofazimine alone and in combinations with pretomanid and bedaquiline. Am. J. Respir. Crit. Care Med..

[19] Imperial MZ (2018). A patient-level pooled analysis of treatment-shortening regimens for drug-susceptible pulmonary tuberculosis. Nat. Med..

[20] Karinja MN, Esterhuizen TM, Friedrich SO, Diacon AH (2015). Sputum volume predicts sputum mycobacterial load during the first 2 weeks of antituberculosis treatment. J. Clin. Microbiol..

[21] Beal, S. L., Sheiner, L. B., Boeckmann, A. J., & Bauer, R. J. (eds) NONMEM 7.3.0 Users Guides. (1989–2013). ICON Development Solutions, Hanover, MD, accessed 8 January 2019. https://nonmem.iconplc.com/nonmem730/Latest_User_Documents/guides/?token=85E0FE2D-1353-11e9-A308-005056911489&html.

[22] Lindbom L, Pihlgren P, Jonsson N (2005). PsN-toolkit—a collection of computer intensive statistical methods for non-linear mixed effect modeling using NONMEM. Comput. Methods Programs Biomed..

[23] Keizer RJ, Karlsson MO, Hooker A (2013). Modeling and simulation workbench for NONMEM: tutorial on Pirana, PsN, and Xpose. CPT Pharmacometr. Syst. Pharmacol..

